# Multi-Population Analysis for Leaf and Neck Blast Reveals Novel Source of Neck Blast Resistance in Rice

**DOI:** 10.3390/plants13172475

**Published:** 2024-09-04

**Authors:** Ashim Debnath, Hage Sumpi, Bharati Lap, Karma L. Bhutia, Abhilash Behera, Wricha Tyagi, Mayank Rai

**Affiliations:** 1 School of Crop Improvement, College of Post Graduate Studies in Agricultural Sciences (CPGSAS), Central Agricultural University (Imphal), Umiam 793103, Meghalaya, India; adebnathagri@gmail.com (A.D.); hsoompi2293@gmail.com (H.S.); bharatilap05@gmail.com (B.L.); klandup@gmail.com (K.L.B.); abhilashb252@gmail.com (A.B.); 2 Department of Genetics and Plant Breeding, Faculty of Agricultural Sciences, Rajiv Gandhi University, Rono Hills, Doimukh 791112, Arunachal Pradesh, India; 3 Post Graduate College of Agriculture, Dr. Rajendra Prasad Central Agricultural University (RPCAU), Samastipur 848125, Bihar, India; 4 Research Program-Accelerated Crop Improvement (ACI), International Crops Research Institute for the Semi-Arid Tropics (ICRISAT), Patancheru 502324, Telangana, India

**Keywords:** *Magnaporthe grisea*, blast resistance, association, marker, genotypes, population

## Abstract

Rice blast is one of the most devastating biotic stresses that limits rice productivity. The North Eastern Hill (NEH) region of India is considered to be one of the primary centres of diversity for both rice and pathotypes of *Magnaporthe grisea*. Therefore, the present study was carried out to elucidate the genetic basis of leaf and neck blast resistance under Meghalaya conditions. A set of 80 diverse genotypes (natural population) and 2 F_2_ populations involving resistant parent, a wildtype landrace, LR 5 (Lal Jangali) and susceptible genotypes Sambha Mahsuri SUB 1 (SMS) and LR 26 (Chakhao Poireiton) were used for association analysis of reported major gene-linked markers with leaf and neck blast resistance to identify major effective genes under local conditions. Genotyping using twenty-five gene-specific markers across diverse genotypes and F_2_ progenies revealed genes *Pi5* and *Pi54* to be associated with leaf blast resistance in all three populations. Genes *Pib* and *qPbm* showed an association with neck blast resistance in both natural and LR 5 × SMS populations. Additionally, a set of 184 genome-wide polymorphic markers (SSRs and SNPs), when applied to F_2_-resistant and F_2_-susceptible DNA bulks derived from LR 5 × LR 26, suggested that *Pi20(t)* on chromosome 12 is one of the major genes imparting disease resistance. Markers snpOS318, RM1337 and RM7102 and RM247 and snpOS316 were associated with leaf blast and neck blast resistance, respectively. The genotypes, markers and genes will help in marker-assisted selection and development of varieties with durable resistance.

## 1. Introduction

Rice (*Oryza sativa* L.) is the primary source of food for a major portion of the world’s population [1] and a source of more than two-thirds of the calorie intake among the poorer populations of tropical and subtropical countries (including the North Eastern Hill (NEH) region of India) [2]. The total yield of the NEH region of India is estimated to be around 15.85 tonnes/ha [3], which is relatively low. Therefore, increasing rice production is crucial to feed the ever-growing world’s population. However, the productivity of rice is adversely affected by various biotic and abiotic factors, with blast disease being a leading cause of crop loss in the NEH region.

Blast disease caused by the filamentous fungus *Magnaporthe grisea* (synonymous with *Pyricularia oryzae*) is one of the serious diseases that causes a severe reduction in rice yield. The pathogen can cause damage up to 90–100% under favourable conditions [4,5]. The fungi can cause leaf blast, nodal blast and neck or panicle blast. Among these, neck blast is considered the most destructive phase of the disease and can occur independently of leaf blast [6]. Neck blast causes direct yield reduction, since grain filling on infected panicles is poor, making it the most serious phase of the blast disease [7]. Even without undergoing sexual reproduction, *M. grisea* is capable of rapid genetic changes through active transposable elements, which can lead to a loss of avirulence (AVR) genes, resulting in the evasion of host defense and the occurrence of rice blast disease. So far, over 100 major blast R genes have been identified, with 37 of them cloned and characterised [8]. Cloning of the dominant gene *Pi-kh* from indica genotype Tetep after mapping is one such example [9]. Additionally, several resistant genes like *Pi9* from *Oryza minuta*, *Pi-40(t)* from *Oryza australiensis* and *Pirf2-1(t)* from *O. rufipogon* have been identified [10]. The *japonica* cultivar Miyazakimochi showed resistance to panicle blast, and introgression of *Pi64* into susceptible cultivars via gene transformation and marker-assisted selection conferred high-level and broad-spectrum leaf and neck blast resistance to *indica*-sourced isolates, demonstrating its potential utility in breeding broad-spectrum resistant rice cultivars [11]. Akhanaphou, a unique rice landrace of Manipur showing a high level of resistance to leaf and neck blast and subsequent genetic characterisation of the QTLs, was identified for resistance and led to the identification of two major genes *Pi38* and *Pitp* [12]. Molecular breeding programs for resistance to blast disease in rice effectively try to manage the disease by integrating the reported resistant genes in popular cultivars lacking them [13]. Compared with leaf blast resistance, our understanding of panicle blast resistance is limited.

**Figure 1:**
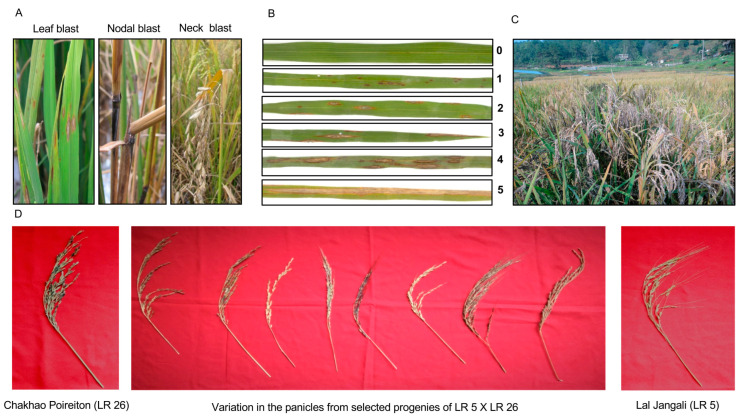
Representative photos showing (**A**) leaf, nodal and neck blast symptoms in experimental plot. (**B**) Differential disease reaction for leaf blast showing disease severity on a scale from 0 to 5. (**C**) Susceptible variety (SMS) showing severe blast in the field conditions. (**D**) Variation in panicle architecture and blast symptoms in selected progenies of biparental cross (LR 5 × LR 26) as compared to parents (Chakhao Poireiton (LR 26)—susceptible; Lal Jangali (LR 5)—resistant).

Northeast India is a centre of diversity for rice. Several wild species and landraces of rice found in this region have been identified for blast resistance, but there is very little or no comprehensive information available regarding major resistant genes effective against blast pathogens of this region. However, the detailed molecular characterisation and utilisation of the landraces imparting resistance against this fungal pathogen is still lacking. Identification of novel R-genes/QTLs or mining the new alleles of blast or R-genes with broad-spectrum resistance and pyramiding different R-genes/QTLs with different resistance spectra into the elite cultivars is the best way to achieve durable and broad-spectrum blast resistance [14]. Therefore, for sustainable production of rice, it is important to continue the search for new resistance sources of the blast and incorporate them into the pre-breeding pipeline. Some of the landraces from the Northeast possessing resistance genes for blast disease include Biran from Tripura state having nineteen *R* genes [15]; Phaudel, Bachithima from Sikkim carrying the desired alleles for reported blast-resistant genes *Piz*, *Pikh*, *Pita* and *Pib* [16]; and Nungshang Phou from Meghalaya containing the *Pi2*, *Pi5*, *Pi54* and *Pib* resistant genes [17]. Our recent study revealed that *Pb1* is associated with leaf blast resistance, suggesting its role in imparting resistance to leaf blast at least under Meghalaya [18]. In addition, we identified a landrace, LR 5 (Lal Jangli), which resembles *O. nivara* (Figure 1) and has shown resistance to leaf and neck blasts across several seasons under Meghalaya conditions. Previously, we had characterised this landrace for two genes, *PSTOL1* and *PupK20-2*, reported for low P tolerance [19].

The current study focuses on the identification of genotypes resistant to leaf and neck blasts and their characterisation using markers reported for genes imparting resistance. Additionally, marker-trait association in a set of NEH rice germplasm and two biparental populations led to the identification of the most effective resistance genes/loci against local pathotypes.

## 2. Results

### 2.1. Phenotypic Variation in Response to Local Blast Pathotypes

Two biparental populations and a natural population comprising 80 genotypes were used in the current study (Table 1; details mentioned in materials and method; Table S1 (from Supplementary File)). Blast disease evaluation for leaf blast score and neck blast percentage for the natural population for both the seasons for the agroecologies (lowland and upland) in normal and late-sown conditions showed variation (Table S2 (from Supplementary File) revealing BAM 5850 as the most susceptible genotype followed by LR 26, BAM 4496, BAM 4168, BAM 8315 and BAM 2680 for leaf blast under lowland conditions. Neck blast infestation was higher for late sown as compared to timely sown condition. In upland conditions (season 2), several monogenic differential lines, BAM lines, UR and JR lines were resistant to leaf blast. Severe neck blast infestation (more than 50% panicles affected) was observed for LR 26, BAM 8381, BAM 7449, IRBLZ5CA, IR2NIR20, BAM 4534, BAM 5850 and IRBL-b-R under timely sown condition, whereas under the late-sown condition, BAM 7449 and LR 26 were severely affected.

**Figure 2:**
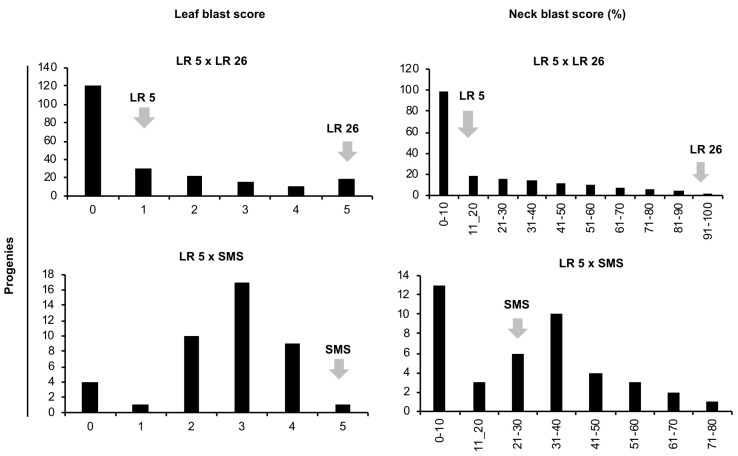
Frequency distribution of genotypes for leaf and neck blast. Leaf blast and neck blast score in F_2_ progenies of two biparental populations, viz., LR 5 × LR 26 and LR 5 × SMS. X-axes represent disease severity score for leaf (0–5 scale; based on lesion length on leaves) and neck blast (percentage (%) of panicles affected by neck blast), respectively. Y-axes represent number of progenies. LR 5—Lal Jangali (resistant landrace); LR 26—Chakhao Poireiton (susceptible genotype); SMS—Sambha Mahasuri SUB1 (susceptible variety).

In the case of F_2_ populations, the frequency distribution of both leaf and neck blast indicated the role of multiple dominant resistance genes (Figure 2).

### 2.2. Genotyping for Reported Genes Conferring Resistance to Blast Disease

In the natural population, for each of the 25 reported markers spread across chromosomes 1, 6, 9, 11 and 12, allelic variation among a diverse set of genotypes was studied (Table S3 (from Supplementary File). Out of the 25 markers used, four alleles each were detected for four markers, viz., JJ803, AP5659, RM224 and RM1337. Marker RM512 was found to be monomorphic, while the highest numbers of heterozygotes were observed for markers RM1337 and MRG4766, respectively.

**Figure 3:**
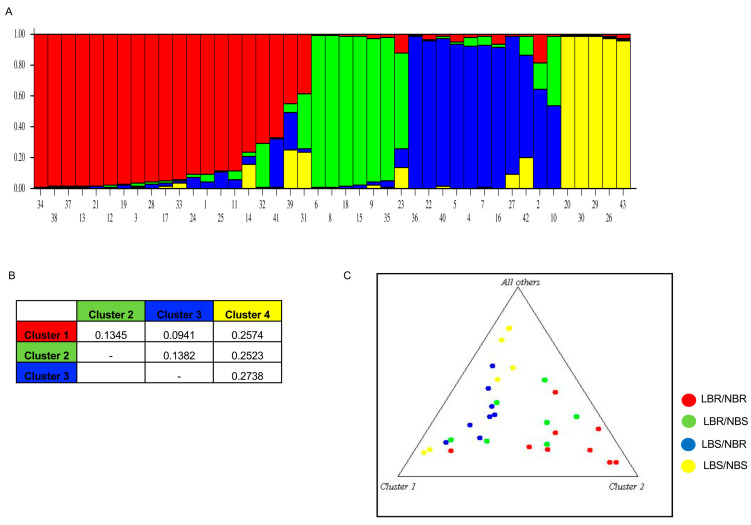
Population structure estimation of the 43 rice lines based on SSR markers with STRUCTURE software output at K = 4. (**A**) Natural population. The serial numbers of genotypes are labelled on the x-axis (Table S5 (from Supplementary File)), while y-axis denotes percentage ancestry. (**B**) Net nucleotide distance between clusters. (**C**) Genotypic clustering of blast resistance and susceptible pools for LR 5 × LR 26. Net nucleotide distance between four clusters computed using point estimates of P. Triangle plot showing genotypic clustering of blast resistance and susceptible pools (LR 5 × LR 26). Leaf blast—LB and neck blast—NB.

The genetic structure analysis using Bayesian STRUCTURE software version 2.3.4 based on identified percentage ancestry (cut off > 80%) in the 43 genotypes revealed 4 distinct genetic clusters. Cluster 1 composed of 15 rice lines, viz., LR 5, SMS, LR 26, BAM 452, BAM 1529, BAM 2815, BAM 3690, BAM 4478, BAM 4496, BAM 4534, BAM 5850, BAM 7385, BAM 7449, BAM 7859 and BAM 8296; 6 genotypes (9W291, 4SHDH14, UR 3, UR 4, LR 11 and JR 54) grouped in cluster 2; 8 genotypes (IR2NIR20, DH1210, IRBL-b-R, JR 53, IRBLKK3, BLZ5CA, IRBL-5-6 and UR 1) in cluster 3 and 5 lines (JR 60, JR 68, JR 69, UR 2 and UR 7) in cluster 4. When the cut-off was lowered to 60%, six more genotypes (JR 19, JR 31, BAM 1659, BAM 5779, BAM 8381 and BAM 74290 could be clustered in the first three clusters (Figure 3A). The rest of the rice genotypes were admixtures based on the reported genic markers. The neck blast score varied in each of the clusters suggesting the possibility of finding genetically distinct sources of neck blast resistance. Cluster 4 having five genotypes had a NB score of 0.

The overall proportion of the membership of the sample in each of the clusters was 0.415, 0.193, 0.247 and 0.146, respectively for the four clusters. Average heterozygosity in the four clusters was 0.3640, 0.2549, 0.4461 and 0.2363, respectively. To explore the genomic distribution of subpopulation differences, we examined F_ST_ values between pairs of subpopulation groups defined at K = 4. The mean F_ST_ values ranged from 0.2773, 0.4988, 0.2240 and 0.6560 for the four clades, respectively. The net nucleotide distance in the four clades varied from 0.094 between clusters 1 and 3 to 0.27 between clusters 3 and 4 (Figure 3B).

In the parental polymorphism survey of LR 5 and SMS, out of 25 markers used, 8 markers, viz., JJ803, S29742, MRG4766, RM7311, PB3810, RM224, Pi54 Indel and RM527 were polymorphic between parents (Table S4 (from Supplementary File)); and for LR 5 and LR 26, 35 markers (16 SSRs and 19 SNPs) were polymorphic from a total of 184 markers surveyed. These polymorphic markers were able to segregate progenies showing resistance to the NBR/LBR cluster separately (Figure 3C).

### 2.3. Marker–Trait Association

**Figure 4:**
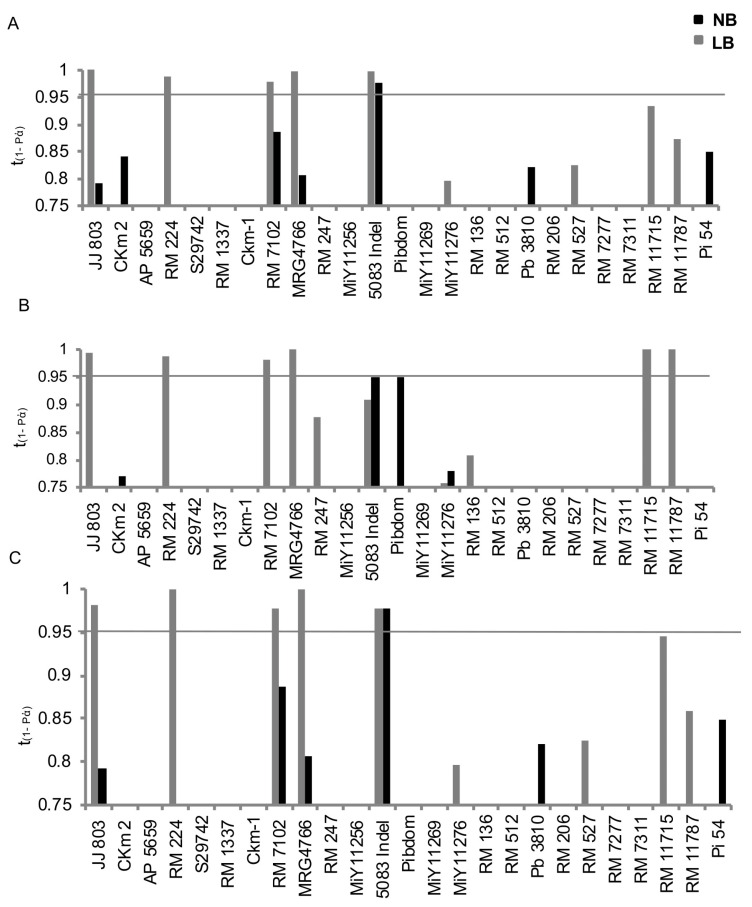
Association of candidate gene-based/-linked markers with leaf blast (LB) and neck blast (NB) resistance. (**A**) Timely sown upland, (**B**) late-sown upland, (**C**) timely sown lowland conditions. The x-axis shows the candidate gene-based/-linked markers (Table S3 (from Supplementary File)). The y-axis represents 1-*P*(α) value for t distribution where *P*(α) is the probability of type-I error. The marker bars above 0.95 on Y-axis show significant association.

Under timely sown upland conditions, five markers (JJ803, RM224, RM7102, MRG4766 and 5083 Indel) were associated with leaf blast resistance. Similarly, under late-sown upland conditions, six markers (JJ803, RM224, RM7102, MRG4766, RM11715 and RM11787) were associated with leaf blast resistance. Marker 5083 Indel was associated with leaf blast resistance under timely sown upland conditions, while four markers were associated with leaf blast resistance both under timely and late-sown lowland conditions (Figure 4). Marker 5083 Indel was significantly associated with neck blast resistance under timely sown upland and lowland conditions. According to the Chi-square test, markers JJ803, RM224 and MRG4766 for SMS/LR 26 allele were associated with leaf blast susceptibility. For the marker RM7102, resistance was associated with LR 26 allele (Table 2). For MRG4766, allele A (JR 60/UR 7) was associated with resistance, whereas for RM7012, allele A (LR 26) was significantly associated with susceptibility. In the case of JJ803, allele D (JR 60/UR 7) was found to be significantly associated with leaf blast resistance and allele C (JR 19) was present in high frequency in the resistant sub-group of RM224. In the case of marker 5083 Indel, allele A (JR 69/UR 7) and B (JR 31) were present only in the resistant sub-group, whereas allele C was equally distributed in both the sub-groups. With respect to neck blast resistance, allele A (JR 60/UR 7) of marker 5083 Indel was present only in the resistant sub-groups.

In the case of F_2_ populations derived from LR 5 × SMS, when dominance allelic interaction was assumed, all eight polymorphic markers were found to be significantly associated with leaf blast resistance. However, when additive allelic interaction was assumed, only PB3810, RM224 and Pi54 Indel were observed to be associated with leaf blast resistance (Table 3). None of the markers were found to be significantly associated with neck blast resistance in the F_2_ population and, interestingly, none were found to be heterozygous for RM527.

**Figure 5:**
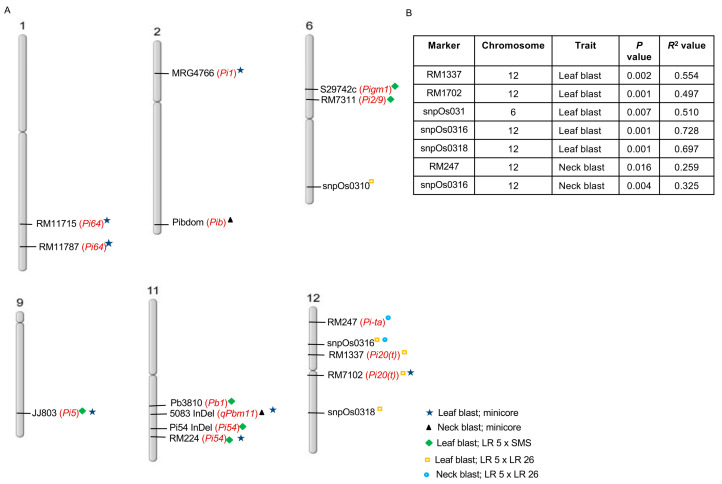
Markers associated with neck blast resistance and leaf blast resistance in three populations. (**A**) Map shows position of reported blast markers associated with three populations. (**B**) Summary of significant markers for LR 5 × LR 26, along with phenotypic percentage variation, explained (*R*^2^ values).

In the case of the F_2_ population derived from LR 5 × LR 26, a total of five markers (RM1337, RM7102, snpOS0310, snpOS0316 and snpOS0318) showed an association for both resistant and susceptible groups in the case of leaf blast (Table 4). For neck blast, two markers (RM247 and snpOs0316) showed association with the two extreme groups. However, two markers (snpOs0310 and snpOs0311) showed a significant association for the susceptible group only in the case of neck blast (Table 4). Marker–trait association-based Chi-square and regression analysis showed RM1337 (*p* value = 0.002 and *R*^2^ = 0.554), RM7102 (*p* value = 0.001 and *R*^2^ = 0.497), snpOS0310 (*p* value = 0.007 and *R*^2^ = 0.510), snpOS0316 (*p* value = 0.001 and *R*^2^ = 0.728) and snpOS0318 (*p* value = 0.001 and R^2^ = 0.697) showed association with resistance and susceptibility against leaf blast, whereas RM247 (*p* value = 0.016 and *R*^2^ = 0.259) and snpOS0316 (*p* value = 0.004 and *R*^2^ = 0.325) showed association with these two groups for neck blast (Figure 5A). Except for snpOS0316, which was present on chromosome 6, the rest of the four markers found significant were located on chromosome 12. The genotype of the progenies with respect to RM1337 could explain 55% of the phenotype, i.e., leaf blast score in extreme bulks. Progenies carrying the LR 5 allele for RM1337 showed a lower blast score (0–2), whereas in the progenies carrying the LR 26 allele, the blast score ranged from 1 to 5. Progeny heterozygous for the marker showed a blast score ranging from (0–3). Similarly, regression for blast resistance with other marker genotypes was also evaluated, where genotype of progenies with respect to RM7102, snpOS0316 and snpOS0318 could explain 49%, 72% and 69% of the leaf blast score in the contrasting bulks (Figure 5B). Markers RM247, snpOS0310 and snpOS0316 could explain 25%, 32% and 38%, respectively, of the neck blast percentage in the bulk with extreme phenotype.

No neck blast was found in progenies carrying the LR 5 allele for snpOS0310 and snpOS0316, whereas the neck blast percentage in the progenies carrying the LR 26 allele ranged from no neck blast to 100% neck blast infection. Neck blast percentage in progenies heterozygous for these two markers also ranged from 0 to 100%. However, RM247 was found to be an exception where no heterozygous was found among the two extreme bulks, and progenies carrying LR 5 allele for RM247 also showed neck blast infection with a neck blast percentage ranging from 60 to 100%.

## 3. Discussion

The North Eastern Hill (NEH) region of India is not only a blast endemic region but is also rich in diverse rice germplasms, which are thought to harbour novel alleles that will provide resistance against local pathotypes of this region. The present study involved three different populations, i.e., one natural population and two F_2_ populations. In both the F_2_ populations, LR 5 was used as a resistant parent because, while phenotyping for other traits [19,20], this genotype consistently showed resistance to blast disease. Marker–trait association studies on natural and F_2_ population derived from LR 5 xSMS revealed an association between seven reported markers linked to seven different loci/genes (*Pi54*, *Pi2*, *Pib*, *Pi2/9* locus, *Pi5*, *Pb1*/qPbm locus and *Pi20(t*)) contributing to leaf and neck blast-resistant. *Pi54*, which was earlier known as *Pi-kh*, is a dominant gene and has been reported to be effective against *M. grisea* populations prevalent in the North Western Himalayan region of India [21]. However, screening of our germplasm revealed that *Pi54* is already present in several varieties and landraces grown in the NEH region, indicating natural selection for the resistant allele. The presence of different blast genes *Pitp*, *Pi33*, *Pi54*, *Pib*, *Pi20*, *Pi38*, *Pita2*, *Pi1*, *Piz*, *Pi9*, *Pizt* and *Pi40* has been previously reported in different landraces of NE India [22]. Our recent work on a biparental population derived from a high-yielding variety Sahbhagi dhan and Chakhao Poireiton (purple coloured local cultivar) revealed that reported blast resistance genes *Pita*, *Piz-5*, *Piz* and *Pb1* were polymorphic between these two genotypes and *Pb1*, reported to be associated with panicle blast resistance, contributed to leaf blast resistance [18].

Genes *Pi5* and *Pi54* were found to be associated with leaf blast resistance in both the natural population and the F_2_ population. Landraces of Manipur harbour the resistant allele for *Pi5*. Genes *Pib* and *qPbm* showed some degree of association with neck blast resistance in the natural population [23]. However, this needs to be validated on a larger population. It has been previously reported that the *Pb1* gene shows substantial resistance to panicle blast isolated and cloned from the *indica* rice cultivar Modan. Promoter:GUS analysis indicated that genome duplication played a crucial role in the generation of *Pb1* by placing a promoter sequence upstream of its coding sequence [24]. qPbm11, a major QTL on chromosome 11, is distinct from *Pb1* and showed resistance to panicle blast [25]. Two quantitative trait loci (QTLs), *qPb11-1*(*Pb-bd1*) and *qPb6-1*, have also been identified from *japonica* landraces Bodao and Suyunuo, respectively, imparting panicle blast resistance [26]. This is primarily because a neck blast is difficult to score and may be governed by multiple genes. Our study also reported markers and genotypes (including landrace like LR 5) resistant to neck blast. However, the association as reflected by *R*^2^ values was not as strong as that for leaf blast. This could be due to other genes reflecting the quasi-quantitative nature of the trait. Additionally, though the *R*^2^ values are small, they are not insignificant and the marker and underlying allele can still be used for MAS in breeding programs.

Out of 184 markers used to detect polymorphism on the parental genotype, 35 markers were polymorphic, which were used for genotyping the selected extreme bulks for cross 2 (LR 5 × LR 26). Frequency distribution analysis for leaf blast score and neck blast percentage revealed that the trait was governed by multiple loci, indicating the role of dominant genes. Marker–trait association-based Chi-square revealed that five markers showed significant association with tolerant and susceptible groups out of which, one was present in chromosome 6 and the rest four markers were present on chromosome 12. The four markers found on chromosome 12 were RM1337, RM7102, snpOS316 and snpOS318. RM1337 and RM7102 were co-localised with the already-reported blast resistance gene *Pi20(t)*. Five *Pi20(t)* markers were previously identified and mapped, suggesting that RM1337 and RM7102 markers present near the centromere region of chromosome 12 co-segregated with the *Pi20(t)* gene, with no recombination being observed between the markers and the target gene [27]. The present study used these two markers, which showed an association with blast resistance. Both of these markers reported to be linked with *Pi20(t)* blast resistance gene can be useful for marker-assisted selection programs.

Insilico approach helped in identifying different putative candidate genes lying between the two polymorphic SSR markers, RM1337 (Os12g03140) and RM7102 (Os12g23520), which were associated with tolerant and susceptible bulks for leaf blast in the present study. More than 100 genes are present between these 2 SSR markers, out of which 7 putative disease resistance genes (including genes with NBS-LRR domain, NB-ARC domain and other disease resistance genes such as stripe rust resistance gene) were identified in the region. Therefore, it is likely that the broad-spectrum resistance exhibited by the *Pi20(t)* gene may be due to the action of the several NBS-LRR genes present between RM1337 and RM7102. With the availability of the whole genome sequence along with the pangenome genome for several rice genotypes, haplotype breeding targeting several desirable alleles simultaneously is now feasible [28]. Marker snpOS318 associated with leaf blast lies in Os12g30440 of the long arm of chromosome 12, which codes for a putative transposon protein, CACTA (En/Spm sub-class).

RM1337 and RM7102 were known to co-localise with the already-reported *Pi20(t)* gene, suggesting its role in resistance to local pathotypes. In the future, more such markers can be used for further mapping of the segment of chromosome 12 in a larger population for blast resistance. The markers and genes identified in this study can be used for marker-assisted selection of breeding material to increase the frequency of favourable alleles at these loci in the elite populations, as it is evident from this study that a single major gene may not be able to provide durable resistance. It may be highlighted that leaf and neck blast resistance is primarily governed by dominant alleles of multiple genes that interact with each other to impart resistance. An effective breeding strategy would involve stacking such major genes as identified in this study in elite backgrounds with the help of Marker-assisted Selection (MAS). However, this study failed to identify effective neck blast-resistant loci that are effective across populations and years. This may suggest the existence of unreported loci for neck blast resistance that may have small additive effects.

## 4. Materials and Methods

### 4.1. Plant Materials

The experiment was conducted at the College of Post Graduate Studies in Agricultural Sciences, CAU (I), Umiam, Meghalaya. A set of 80 rice genotypes comprising lines from Indian rice mini core collection [29,30], local cultivars from upland, lowland and jhum ecosystems [31], advanced breeding lines, and fourteen monogenic differentials (IRBL) [32,33] were screened across 2 seasons, under 2 different rice ecosystems (upland and lowland) (Table S1 (from Supplementary File)). The 80 genotypes were grown in both upland (direct seeded) and lowland (transplanted) conditions in augmented field design at 2 different sowing dates—timely and late (a gap of 20 days in sowing)—with a spacing of 20 cm between rows and 15 cm between plants. Ten plants per line and Chakhao Poireiton (LR 26) in between the lines were planted. LR 26 is a popular local black sticky rice genotype from Manipur [18,19,20], which is blast-susceptible, and was therefore used as a susceptible check. Simultaneously, two biparental populations were generated, with LR 5 as the common blast-resistant parent (Table 1). Sambha Mahsuri SUB1 (SMS), a mega variety [34], is blast-susceptible. Progenies of both the biparental F_2_ crosses were grown under lowland conditions with a spacing of 20 cm × 20 cm between rows and plants.

### 4.2. Disease Scoring and Genotyping

Leaf and neck blast were scored separately under natural field conditions at three different time intervals of 30, 60 and 90 days after transplanting. Leaf blast disease was scored according to the method described by Mackill and Bonman [35], with the score ranging from 0 (zero = no lesions) to 5 (five = severely affected plant) (Figure 1). In the case of neck blast, the disease was scored based on the percentage of infected panicles. Genotypes with 0–20% infected panicles were considered resistant and genotypes with 50% and more infected panicles were considered susceptible.

Bulk segregant analysis (BSA) was used for genotyping. In the case of the natural population, a subset (43 genotypes out of 80) consisting of both leaf and neck blast-resistant and susceptible genotypes based on the two-season field experiments was made (Table S2 (from Supplementary File)). Based on disease score across seasons, ecosystems and sowing time, the natural population, consisting of 43 genotypes, along with 42 and 29 F_2_ progenies from the crosses of LR 5 × SMS and LR 5 × LR 26, respectively, were selected, representing the two extreme groups (resistant and susceptible) for further molecular analysis.

Leaf samples were collected from the genotypes after 30 days of transplanting. Plant genomic DNA was extracted using the CTAB (Cetyl-Tri methyl Ammonium Bromide) extraction method [36]. Genotyping was performed using (a) markers reported for blast resistance located on six different chromosomes (1, 2, 6, 9, 11 and 12) and (b) chromosome-wide markers. While a set of twenty-five SSR/gene-based markers reported for blast resistance located on six different chromosomes were surveyed on all three populations (Table S3 (from Supplementary File)), only LR 5 × LR 26 F_2_ progenies were assessed with genome-wide markers. A set of 184 markers including genome-wide SSRs [37] and 34 SNPs (trait-based SNP panels available at IRRI) [30] were surveyed for polymorphism for this cross. Polymerase Chain Reaction (PCR) was carried out using thermo scientific thermocycler as follows: approximately 50–70 ng template DNA with 5.45 µL sterile distilled water, 1X PCR buffer, 1.75 mM MgCl_2_, 0.12 mM dNTPs, 1.2 µM primer and 0.25 U Taq polymerase in a final volume of 10 µL were used to run the PCR reaction for 33 cycles. The PCR products were analysed by electrophoresis using an agarose gel (1.5–3%) and the gels visualised in a UV gel documentation system (AlphaImager Mini).

### 4.3. Allelic Score and STRUCTURE Analysis

The amplified PCR products subjected to gel electrophoresis were subsequently scored. The population structure analysis of the diverse genotypes and F_2_ progenies based on polymorphic markers was investigated using STRUCTURE version 2.3.4 [38]. The number of subpopulations (K) was estimated using the programme at different K values by setting at K = 1 to 10, with 5 independent iterations per K using the admixture model and allele frequencies correlated. Each run was based on 200,000 Markov chain Monte Carlo (MCMC) iterations after the 200,000 burn-in phase. The delta K (*ΔK*) value was estimated to determine the most likely *K*-value using STRUCTURE HARVESTER software version 0.6.93 [39].

### 4.4. Marker–Trait Analysis

The frequency distribution of F_2_ progenies was evaluated and simple regression analysis using the linear regression model for testing the association between the genotypic classes and the phenotype, and to compare and understand the relationship between marker, genotypes and blast severity, was conducted. In both sets of mapping populations, a *t*-test was used to test the significance of the difference observed between marker classes with respect to leaf and neck blast resistance. A significant *t* test (1-*P* (α) ≥ 0.95) indicated a marker–trait association and the coefficient of determination (*R*^2^) denoted the percentage of variation explained. A Chi-square test for goodness of fit was carried out to test the deviation of the allelic frequencies observed in the tolerant and susceptible bulks as against the respective overall frequencies (expected) in the F_2_ individuals genotyped.

## 5. Conclusions

In conclusion, we report LR 5 as a novel resistant donor harbouring a unique set of alleles for genes already reported for blast resistance (both leaf and neck blast). Additionally, other potential donors (JR 60, JR 68, JR 69, UR 2 and UR 7) for neck blast have also been identified. The marker–trait association suggests that *Pi20(t)* on chromosome 12 is one of the major genes imparting disease resistance in LR 5 × LR 26 biparental cross with markers snpOS318, RM1337 and RM7102 and RM247 and snpOS316 associated with leaf blast and neck blast resistance, respectively. The genotypes, markers and genes will help in marker-assisted selection and the development of varieties with durable resistance.

## Figures and Tables

**Table 1:** Number of extreme genotypes/lines/plants selected based on disease phenotype along with number of markers used for genotyping in marker trait association study.

Population	Crosses	No. of Plants/Genotypes/Lines	Markers
Natural population	-	43 genotypes	25
Biparental population	LR 5 × SMS	42 plants	25
	LR 5 × LR 26	29 plants	184

SMS—Sambha Mahsuri SUB1 (susceptible variety); LR 5—Lal Jangli (resistant landrace); LR 26—Chakhao Poireiton (susceptible genotype).

**Table 2:** Chi-square test for detecting significant deviation in allelic frequencies of candidate gene-linked markers in leaf and neck blast resistant and susceptible groups for the natural population.

Markers	Leaf Blast (χ^2^ Value)		Neck Blast (χ^2^ Value)	
	Resistance	Susceptible	Resistance	Susceptible
JJ803	0.001 *	0.000 *	0.763	0.525
Ckm2	0.315	0.077	0.735	0.482
AP5659	0.461	0.174	0.866	0.704
RM224	0.175	0.023 *	0.959	0.899
S29742	0.55	0.255	0.990	0.974
RM1337	0.341	0.091	0.761	0.522
Ckm-1	0.975	0.936	0.786	0.562
RM7102	0.227	0.039 *	0.889	0.988
MRG4766	0.109	0.009 *	0.927	0.826
RM247	0.826	0.631	0.997	0.992
5083InDel	0.152	0.017 *	0.297	0.068
MiY11269	0.625	0.336	1.000	1.000
MiY11276	0.273	0.057	0.527	0.231
RM136	0.728	0.472	0.997	0.992
RM512	0.995	0.988	0.933	0.841
Pb3810	0.937	0.848	0.821	0.622
RM206	0.562	0.266	0.998	0.995
RM527	0.332	0.086	0.738	0.487
RM7311	0.997	0.991	0.933	0.840
RM11715	0.571	0.276	0.643	0.357
RM11787	0.740	0.491	0.770	0.536
Pi54 InDel	0.982	0.953	0.500	0.207

For each marker, allelic frequencies in resistant and susceptible subgroups were tested against their overall frequency in 43 genotypes for goodness of fit. Alleles that had Chi-square *P*α values of less than 0.05 were considered to be associated with the trait. Asterisk mark indicates significant association of the marker with resistance and susceptibility.

**Table 3:** Value of *t*-test for testing the significance of differences between different genotypic classes with respect to leaf and neck blast score as observed in F_2_ population of LR 5 × SMS. Asterisk mark * and ** indicate significant association of the marker at 5% and 1% level of significance, respectively.

Marker	Mean (AA)	Mean (AB)	Mean (BB)	t (Additive)	t (Dominance)
	Leaf Blast Score				
JJ803	2.846	2.533	2.636	0.410	3.588 **
S29742	2.947	2.083	2.571	0.807	4.052 **
MRG4766	3.333	2.450	1.500	2.277	2.236 *
RM7311	3.111	2.250	2.400	1.553	3.325 **
PB3810	3.375	2.769	1.167	3.973 **	2.446 *
RM224	3.571	2.500	1.429	3.790 **	3.371 **
Pi54	3.600	2.643	1.250	3.287 **	2.777 **
RM527	2.700		2.500	0.448	
**Marker**	**Neck Blast Score**				
	**Mean (AA)**	**Mean (AB)**	**Mean (BB)**	**t (Additive)**	**t (Dominance)**
JJ803	12.978	19.247	23.279	1.273	1.429
S29742	12.407	21.885	21.885	1.517	1.179
MRG4766	14.250	23.067	27.020	1.049	1.032
RM7311	15.344	21.675	34.846	1.703	1.908
PB3810	12.349	23.736	28.287	1.879	1.168
RM224	18.866	22.687	27.024	0.830	1.537
Pi54	10.222	26.948	27.260	1.649	0.679
RM527	15.376		26.134	1.717	

**Table 4:** Chi-square test for goodness of fit to test the observed allelic frequencies in resistance and susceptible groups against the overall frequency of alleles (expected) in the 29 F_2_ progenies (LR 5 × LR 26) genotyped.

Markers	Leaf Blast (χ^2^ Value)		Neck Blast (χ^2^ Value)	
	Resistance	Susceptible	Resistance	Susceptible
HvSSR02-14	0.206	0.145	0.140	0.126
HvSSR02-82	0.421	0.372	0.184	0.169
HvSSR03-71	0.722	0.691	0.722	0.691
HvSSR05-59	0.128	0.091	0.371	0.354
S29742 indel	0.440	0.423	0.352	0.315
HvSSR07-38	0.128	0.091	0.371	0.354
JJ803	0.510	0.447	0.480	0.448
HvSSR10-30	0.478	0.431	0.934	0.931
HvSSR11-13	0.196	0.165	0.593	0.565
HvSSR11-23	0.359	0.269	0.845	0.839
RM224	0.699	0.665	0.245	0.194
MiY11276	0.465	0.433	0.257	0.257
RM1337	0.003 *	0.002 *	0.309	0.309
RM247	0.194	0.134	0.025 *	0.016 *
RM7102	0.002 *	0.001 *	0.487	0.480
snpOS0296	0.765	0.726	0.372	0.227
snpOS0298	0.394	0.317	0.315	0.174
snpOS0307	0.890	0.872	0.900	0.864
snpOS0309	0.890	0.872	0.960	0.945
snpOS0312	0.754	0.729	0.864	0.801
snpOS0299	0.612	0.583	0.884	0.831
snpOS0301	0.966	0.962	0.225	0.074
snpOS0302	1.000	1.000	0.117	0.021 *
snpOS0305	0.776	0.745	0.400	0.255
snpOS0308	0.910	0.894	0.464	0.321
snpOS0310	0.020 *	0.007 *	0.098	0.019 *
snpOS0311	0.068	0.068	0.064	0.009 *
snpOS0315	1.000	1.000	0.737	0.628
snpOS0316	0.001 *	0.000 *	0.050	0.004 *
snpOS0318	0.002 *	0.001 *	0.267	0.182
snpOS0022 (BADH2)	0.328	0.279	0.844	0.801
snpOS0024 (CHALK5)	0.591	0.562	0.524	0.404
snpOS0068 (BPH17)	0.441	0.394	0.505	0.382

Chi-square probability values of less than 0.05 (denoted by *) for tolerant/susceptible groups suggested significant deviation of allelic frequencies in the respective groups as compared to overall populations.

## Data Availability

Data are contained within the article and Supplementary Materials.

## Supplementary Materials

The following supporting information can be downloaded at: https://www.mdpi.com/article/10.3390/plants13172475/s1, Table S1: list of genotypes used in the study. Table S2: leaf and neck blast scores observed for genotypes screened under timely sown and late-sown lowland and upland field conditions during kharif (season 1 and season 2). Table S3: list of genes reported for conferring blast resistance targeted for molecular characterisation in the current study. Table S4: genotyping score for LR 5 × SMS. Table S5: leaf and neck blast scores for the panel of rice genotypes. References [40,41,42,43,44,45,46,47,48,49,50,51,52,53,54,55,56,57] are cited in the supplementary materials.
