# Supplementary material for: Multi-Population Analysis for Leaf and Neck Blast Reveals Novel Source of Neck Blast Resistance in Rice
Source: Plants (Basel). 2024 Sep 4;13(17):2475. doi: 10.3390/plants13172475 (PMC11397284; doi:10.3390/plants13172475)
Supplement: Supplementary file 1 [file plants-13-02475-s001.zip › plants-3088091-supplementary.pdf]

Multi-Population Analysis for Leaf and Neck Blast Reveals Novel Source of Neck Blast Resistance in Rice

Ashim Debnath [1,2,†], Hage Sumpi [1,†], Bharati Lap [1,2], Karma L. Bhutia [1,3], Abhilash Behera [1], Wricha Tyagi [1,4,*] and Mayank Rai [1,3,*]

[1] School of Crop Improvement, College of Post Graduate Studies in Agricultural Sciences (CPGSAS), Central Agricultural University (Imphal), Umiam 793103, Meghalaya, India; adebnathagri@gmail.com (A.D.); hsoompi2293@gmail.com (H.S.); bharatilap05@gmail.com (B.L.); klandup@gmail.com (K.L.B.); abhilashb252@gmail.com (A.B.)

[2] Department of Genetics and Plant Breeding, Faculty of Agricultural Sciences, Rajiv Gandhi University, Rono Hills, Doimukh 791112, Arunachal Pradesh, India

[3] Post Graduate College of Agriculture, Dr. Rajendra Prasad Central Agricultural University (RPCAU), Samastipur 848125, Bihar, India

[4] Research Program-Accelerated Crop Improvement (ACI), International Crops Research Institute for the Semi-Arid Tropics (ICRISAT), Patancheru 502324, Telangana, India

* Correspondence: wricha.tyagi@icrisat.org (W.T.); mayank.rai@rpcau.ac.in (M.R.)

† These authors contributed equally to this work.

Supplementary Files

Table S1. List of genotypes used in the study.

S. No.	Genotype Name	S.No.	Genotype Name	S.No.	Genotype Name
1	LR 11	28	IRBL 6	55	BAM 758
2	LR 5	29	IRBL2T	56	BAM 759
3	LR 26	30	IRBLTT	57	BAM 766
4	SMS	31	IRBL25CA	58	BAM 4496
5	CAU R1	32	IRBL-LT	59	BAM 8296
6	LR 23	33	IRBLTACPi	60	BAM 5779
7	LR 5	34	IRBL-b-R	61	BAM 785
8	UR 7	35	IRBLK5F5	62	BAM 812
9	UR 3	36	IRBL-5-6	63	BAM 4510
10	UR 2	37	IRBLTA-LT2	64	BAM 6921
11	UR 1	38	IRBL5M	65	BAM 4408
12	UR 5	39	IRBL K4K3	66	BAM 2680
13	UR 84	40	BAM 452	67	BAM 4168
14	IR2NIR20	41	BAM 1659	68	BAM 1529
15	3SH9W29-1	42	BAM 4168	69	BAM 1108
16	4SHDH140-7-10-3	43	BAM 7385	70	BAM 1057
17	4SHDH140-5-11	44	BAM 2680	71	BAM 2815
18	2CRDH 12-10	45	BAM 5850	72	BAM 2986
19	2CRDH 11-19	46	BAM 1098	73	BAM 1035
20	JR 53	47	BAM 1264	74	BAM 3690
21	JR 19	48	BAM 4534	75	BAM 5834
22	JR 60	49	BAM 8381	76	BAM 7859
23	JR 31	50	BAM 8315	77	BAM 4478
24	JR 69	51	BAM 1659	78	BAM 7449
25	JR 68	52	BAM 4797	79	BAM 2319
26	JR 54	53	BAM 56	80	BAM 7429
27	IRBL2FU	54	BAM 8305		

LR-Lowland Rice; JR–Jhum Rice; SMS-Sambha Mahsuri SUB1; BAM–Bio prospecting & allele mining mini core collection; UR-Upland Rice; IRBL-International Rice Blast nursery lines; CRDH-Introgression lines in the background of CAUR1; SHDH-Introgression lines in the background of Sasharang; CAUR1 (Tamphaphou) - Central Agricultural University Rice variety

Table S2. Leaf and neck blast scores observed for genotypes screened under timely sown and late sown lowland field conditions during kharif (season 1 and season 2).

Leaf blast scores observed for genotypes screened under lowland field conditions during kharif (season 1)

Blast score for timely sown	Genotypes
0	JR 19, IR2NIR20, IRBLTA-LT2, IRBL2FU, IRBL-b-R, LR 23, IRBLTT, JR 54, BAM 8296, BAM-5779, LR 11, IRBLK5F5, IRBL6, IRBLZ5CA, LR 5, UR 7, CAU R1, UR 2, BAM 4797, UR 3, IRBL5-6, BAM 1529, IRBLTACPi, UR 1, IRBL-LT, BAM 1264, BAM 56, BAM 785, BAM 812, BAM 4510, BAM 758, BAM 759, BAM 766, BAM 8305, BAM 1057, BAM 2986, BAM 1035, BAM 5834, BAM 1098
1	SMS, BAM 3690
2	BAM 2319, JR 53, BAM 2815, BAM 4408, IRBLZTT, BAM 6921, BAM 1659
3	IRBLKHK3, IRBL5M, BAM 2680
4	LR 26, BAM 4496, BAM 4168, BAM 8315
5	BAM 5850
**Blast score for late sown**	**Genotypes**
0	JR 19, IR2NIR20, BAM 2319, IRBLTA-LT2, IRBL2FU, IRBL-b-R, LR 23, JR 54, BAM 8296, BAM 5779, JR 53, BAM 4496, LR 11, IRBLK5F5, BAM 3690, IRBL-6, IRBL KHK3, BAM-2815, IRBL Z5CA, BAM-4408, LR 5, UR 7, CAU R1, IRBL5M, IRBLZTT, UR 2, BAM 4797, UR 3, IRBL-5-6, BAM 1529, BAM 8315, IRBLTACPi, BAM 6921, UR 1, BAM 1659, IRBL-LT, BAM 1264, BAM 56, BAM 785, BAM 812, BAM 4510, BAM 758, BAM 759, BAM 766, BAM 8305, BAM 1057, BAM 2986, BAM 1035, BAM 5834, BAM 1098
1	SMS
2	BAM 4168
3	LR 26, IRBLTT, BAM 2680
4	BAM 5850

Leaf blast scores observed for genotypes screened under upland field conditions during kharif (season 2)

Blast Score for timely sown	Genotypes
0	IR2NIR20, BAM 2319, IRBLTA-LT2, 2CRDH12-10, IRBL2FU, 3SH9W29-1, IRBL-b-R, IRBL-TT, 2CRDH-11-19, JR 54,4SHDH140-7-10-3, BAM 8296, UR 4, IRBL K5F5, IRBL 6, JR 69, BAM 4168, IRBLKHK3, JR 31, UR 5, BAM 2815, JR 68, LR 5, UR 7, CAU R1, IRBL5M, IRBLZT-T, UR 2, UR 3, IRBL-5-6, BAM 2680, BAM 8315, IRBLTACPi, BAM 6921, UR 84, UR 1, BAM 1659, JR 60
1	LR 11, BAM 5779, JR19
3	BAM 1108, LR-23, 4SHDH140-5-11, JR 53, BAM 4408, SMS, BAM7859, BAM 7429
4	BAM 4534, BAM 452, BAM 4496, IRBLZ5CA, BAM 8381, BAM 7385, BAM 1529
5	LR 26, BAM 4478, BAM 3690, BAM 5850, BAM 7449, BAM 4408, BAM 4797
Blast Score for late sown	Genotypes
0	IR2NIR20, BAM 2319, IRBLTA-LT2, 2CRDH12-10, IRBL2FU, 3SH9W29-1, IRBL-b-R, LR 23, IRBL-TT, 2CRDH-11-19, JR 54, 4SHDH140-7-10-3, BAM 8296, UR 4, JR 69, IRBLK4K3, JR 31, UR 5, JR 68, IRBL25CA, UR 7, CAUR1, IRBL5M, IRBLZTT, UR 2, BAM 4797, BAM 8381, UR 3, IRBL-5-6, BAM 2680, BAM 1529, BAM 8315, IRBLTACPi, BAM 6921, BAM 7859, UR 84, UR 1, BAM 1659, JR 60
1	LR 5, LR 11, BAM 5779, JR19
2	BAM 1108, 4SHDH140-5-11, BAM 452, BAM 3690
3	BAM 4534, JR 53, IRBL K5F5, BAM 4408, BAM 4168, BAM 7449, BAM 2815, BAM 4408
4	BAM 4478, BAM 4496, BAM 5850, BAM 7385, BAM 7429
5	LR 26, SMS

Neck blast scores observed for genotypes screened under upland field conditions during kharif (season 1)

Neck blast (%) for timely sown	Genotypes
0-10	JR19, BAM 1108, BAM 2319, IRBLTA-LT2, 2CRDH12-10, IRBL-TT, 2CRDH-11-19, BAM 8296, BAM 452, BAM 4478, BAM 5779, UR 04, JR 53, BAM 4496, LR 11, IRBL K5F5, BAM 3690, IRBL 6, BAM 4408, JR 69, BAM 4168, IRBLKHK3, UR 05, BAM 2815, JR 68, LR 5, UR 7, CAU R1, IRBL5M, IRBLZT-T, UR 2, BAM 7385, UR 3, BAM 2680, BAM 1529, BAM 8315, IRBLTACPi, BAM 6921, UR 84, UR 1, BAM 1659, JR 60, 3SH9W29-1, 4SHDH140-7-10-3, IRBL2FU
10-20	SMS, IRBL-5-6, JR 54
20-30	4SHDH140-5-11, BAM 4408
30-40	BAM 4797, JR 31, LR 23, BAM 7429
40-50	BAM 7859
50-60	BAM 5850, IRBL-b-R
60-70	BAM 4534
70-80	IR2NIR20
80-90	BAM 7449, IRBLZ5CA
90-100	LR 26, BAM 8381
**Neckblast (%) for late sown**	**Genotypes**
0-10	JR 19, BAM 1108, BAM 2319, IRBLTA-LT2, 2CRDH12-10, 3SH9W29-1, IRBL-b-R, LR-23, 4SHDH140-5-11, IRBL-TT, 2CRDH-11-19, JR-54, 4SHDH140-7-10-3, BAM 8296, BAM 452, BAM 4478, BAM 5779, UR 04, JR 53, BAM 4496, LR 11, IRBL K5F5, BAM 3690, BAM 4408, JR 69, BAM 4168, BAM 5850, IRBLK4K3, JR 31, UR 05, BAM 2815, JR 68, BAM 4408, LR 05, UR 07, CAU R1, IRBL5M, IRBLZTT, UR 02, BAM 4797, BAM 8381, BAM 7385, UR 03, IRBL-5-6, BAM 2680, BAM 1529, BAM 8315, IRBLTACPi, BAM 6921, BAM 7859, UR 84, UR 01, BAM 7429, BAM 1659, JR 60, BAM 4534
10-20	IRBL2FU
20-30	SMS
30-40	
40-50	IR2NIR20, IRBLZ5CA
50-60	
60-70	BAM 7449
70-80	
80-90	
90-100	LR 26

Neck blast percentage observed for genotypes screened under lowland field conditions during kharif (season 2)

Neck blast (%) for timely sown	Genotypes
0-10	BAM 2319, BAM 8296, LR 11, BAM 3690, IRBL6, BAM 2815, IRBLZ5CA, LR 5, UR 7, BAM 4797, SMS, IRBL-5-6, BAM 2680, BAM 1529, BAM 1659, IRBL-LT, BAM 8305, BAM 1035, BAM 5834
10-20	JR 53, BAM 5779, IRBL KHK3, BAM 1098
20-30	CAU R1, UR 01, IRBLZTT, BAM 6921, IRBLTT, JR 54, BAM 4496, BAM 56
30-40	IRBL-b-R, BAM 8315, LR 23, BAM 759, BAM 1057, JR 19, IRBL2FU, BAM 4408, IRBLTA-LT2, UR 2, BAM 1264, BAM 4510
40-50	IRBLTACPi, BAM 2986, IRBLK5F5, BAM 812, BAM 4168, BAM 785, BAM 5850, BAM 758
50-60	IR2NIR20, IRBL5M, UR 3, BAM 766
60-70	Nil
70-80	Nil
80-90	LR 26
**Neck blast (%) for late sown**	**Genotypes**
0-10	BAM 2319, JR 54, BAM 8296, BAM 5779, JR 53, LR 11, IRBL KHK3, BAM 2815, BAM 4408, LR 5, UR 7, IRBL5M, BAM 4797, SMS, IRBL-5-6, BAM 2680, BAM 1529, IRBLTACPi, BAM 6921, BAM 1659, IRBL-LT, BAM 2986, BAM 5834
10-20	Nil
20-30	Nil
30-40	BAM 8315, LR 23, UR 2, BAM 4496, IRBLZTT, BAM 766, IRBL-6, IRBL2FU, BAM 4168, BAM 1057, BAM 1098
40-50	UR-3, JR-19, BAM-1035, CAU R1, IRBLK5F5, BAM-759, IRBLTT, BAM 3690, BAM 8305
50-60	BAM 56, BAM 758, BAM 4510, IRBL Z5CA, UR 1, BAM1264
60-70	BAM 812, BAM 785, IR2NIR20, IRBLTA-LT2, BAM 5850
70-80	IRBL-b-R
80-90	LR 26

Table S3. List of genes reported for conferring blast resistance targeted for molecular characterization in the current study.

Gene	Chr. No.	Markers	Donor rice variety	Reference
Pi64	1	RM11715, RM1178	Japonica landrace Yangmaogu (YMG)	(Ma et al, 2015)
Pib	2	Pibdom	BL1/Koshihikari	(Wang et al, 1999; Hayashi et al, 2006)
Pi1	2	MRG4766	LAC23	(Inukai et al, 1994; Li et al, 2012)
Pigm(t)	6	S29742c InDel	Gumei4	(Deng et al, 2006)
Pi9	6	RM136	Cultivar TP309	(Qu et al, 2006; Koide et al, 2013)
Piz5	6	RM527	C101A51_CO39	(Deng et al, 2006)
Piz/Piz/Piz 5	6	AP5656-5	Zenith	(Hayashi et al, 2006; Wang et al, 2012)
Pi2/9	6	RM7311	Tianjingyeshengdao	(Qu et al, 2006)
Pi5	9	JJ803	RIL260 (Moroberekan)	(Kwon et al, 2008)
qPbm11	11	Miy11276, Miy11269, Miy11256, 5083 InDel	Miyazaki-mochi	(Ishihara et al, 2014)
Pb1	11	Pb3810, RM7277	Modan	(Fuente et al, 2008; Fujii et al, 2000)
Pi54	11	RM224, Pi54 InDel	Tetep	(Wu et al, 2013)
Pikm	11	Ckm1, Ckm2	Tsuyuake	(Sun et al, 2013; Kaji and Ogawa, 1996; Wang et al, 2007)
Pi38	11	RM206	CO39 and Tadukan	(Gowda et al, 2006)
Pita	12	RM247	Yashiro-mochi and Tsuyuake/Tadukan (I)	(Hayashi et al, 2006)
Pi12	12	RM512	Hong-jiaozhan/Moroberekan (J)	(Inukai et al,1996)
Pi20(t)	12	RM7102, RM1337	Asominori and IR24	(Liu et al, 2007; Liu et al, 2008)

References

- Li, J., Li, D., Sun, Y. and Xu, M. (2012). Rice blast resistance gene Pi1 Identified by MRG4766 marker in 173 Yunnan rice landraces. Rice Genomics and Genetics 3(1).
- Ishihara, T., Hayano-Saito, Y., Oide, S., Ebana, K., La, N.T., Hayashi, K., Ashizawa, T., Suzuki, F. and Koizumi, S. (2014). Quantitative trait locus analysis of resistance to panicle blast in the rice cultivar Miyazakimochi. Rice 7(1): 1-11.

- Ma, J., Lei, C., Xu, X., Hao, K., Wang, J., Cheng, Z., Ma, X., Ma, J., Zhou, K., Zhang, X. and Guo, X. (2015). Pi64, Encoding a Novel CC-NBS-LRR Protein, Confers Resistance to Leaf and Neck Blast in Rice. The American Phytopathological Society, 28(5): 558–568.
- Wang, Z.X., Yano, M., Yamanouchi, U., Iwamoto, M., Monna, L., Hayasaka, H., and Sasaki, T. (1999). The Pib gene for rice blast resistance belongs to the nucleotide binding and leucine-rich repeat class of plant disease resistance genes. Plant J. 19(1): 55–64.
- Hayashi, K., Yoshida, H., and Ashikawa, I. (2006). Development of PCR-based allelespecific and InDel marker sets for nine rice blast resistance genes. Theor. Appl. Genet. 113 (2): 251–260.
- Inukai, T. and Nelson, R. (1994). Mapping for blast resistance gene H-3 derived from rice cuhivarPai-Kan-Tao. Rep Hokkaido Br Crop Sol See Japan Jap Soc Breed. 35: 54–5.
- Deng, Y., Zhu, X., Shen, Y., and He, Z. (2006). Genetic characterization and fine mapping of the blast resistance locus Pigm (t) tightly linked to Pi2 and Pi9 in a broad-spectrum resistant Chinese variety. Theor. Appl. Genet. 113(4): 705–713.
- Wang, Y., Wang, D., Deng, X., Liu, J., Sun, P., Liu, Y., Huang, H., Jiang, N., Kang, H., Ning, Y. and Wang, Z. (2012). Molecular mapping of the blast resistance genes Pi2-1 and Pi51 (t) in the durably resistant rice 'Tianjingyeshengdao'. Phytopathology 102: 779–86.
- Qu, S., Liu, G., Zhou, B., Bellizzi, M., Zeng, L., Dai, L., and Wang, G. L. (2006). The broad-spectrum blast resistance gene Pi9 encodes a nucleotide binding site– leucine-rich repeat protein and is a member of a multigene family in rice. Genetics 172(3): 1901–1914.
- Kwon, S. W., Cho, Y. C., Kim, Y. G., Suh, J. P., Jeung, J. U., Roh, J. H., and Lee, Y. T. (2008). Development of near isogenic japonica rice lines with enhanced resistance to Magnaporthegrisea. Mol. Cells 25(3): 407–416.
- Fuentes, J. L., Correa-Victoria, F. J., Escobar, F., Prado, G., Aricapa, G., Duque, M. C. and Tohme, J. (2008). Identification of microsatellite markers linked to the blast resistance gene Pi-1 (t)in rice. Euphytica 160: 295–304.
- Fujii, H. Y., Saito, K., Sugiura, N., Hayashi, N., Tsuji, T. and Izawa, T. I. M. (2000). Identification of a RFLP marker tightly linked to the panicle blast resistance gene, Pb1, in rice. Breed. Sci. 50 (3): 183–188.
- Wu, Y., Bao, Y., Xie, L., Su, Y., Chu, R., He, W., Huang, J., Wang, J. and Zhang, H. (2013). Fine mapping and identification of blast resistance gene Pi-hk1 in a broad-spectrum resistant japonica rice landrace. Phytopathology 103: 1162–1168.
- Sun, P., Liu, J., Wang, Y., Jiang, N., Wang, S., Dai, Y., Gao, J., Li, Z., Pan, S., Wang, D. and Li, W. (2013). Molecular mapping of the blast resistance gene Pi49 in the durably resistant rice cultivar Mowanggu. Euphytica 192: 45–54.
- Kaji, R., and Ogawa, T. (1996). RFLP mapping of blast resistance gene Pi-km in rice. Int. Rice. Res. Notes 21.
- Wang, Z., Jia, Y., Rutger, J. and Xia, Y. (2007). Rapid survey for presence of a blast resistance gene Pi-ta in rice cultivars using the dominant DNA mar-kers derived from portions of the Pi-ta gene. Plant Breed. 126(1): 36–42.
- Gowda, M., Roy-Barman, S. and Chattoo, B. (2006). Molecular mapping of a novel blast resistance gene Pi38 in rice using SSLP and AFLP markers. Plant Breed. 125(6): 596–599.

- Hayashi, K., Yoshida, H., and Ashikawa, I. (2006). Development of PCR-based allelespecific and InDel marker sets for nine rice blast resistance genes. Theor. Appl. Genet. 113 (2): 251–260.
- Inukai, T., Nelson, R., Zeigler, R., Sarkarung, S., Mackill, D., Bonman, J. and Kinoshita,T. (1996). Genetic analysis of blast resistance in tropical rice cultivars using near isogenic lines, In: G.S. Khush (Ed.), Rice Genetics III. Proc 3rd Int Rice Genet Symp, Oct. 16–20 1995,Manila, The Philippines, 447–450.
- Liu, X., Lin, F., Wang, L. and Pan, Q. (2007). The in-silico map-based cloning of Pi36, a rice coiled-coil-nucleotide-binding site-leucine-rich repeat gene that confers race-specific resistance to the blast fungus. Genetics 176: 2541–2549.
- Liu, W. G., Jin, S. J., Zhu, X. Y., Wang, F., Li, J. H., Liu, Z. R., and Liu, Y. B. (2008). Improving blast resistance of a thermo-sensitive genic male sterile rice line GD- 8S by molecular marker-assisted selection. Rice Sci. 15(3): 179–185.

Table S4 Genotyping score for LR 5 x SMS

S.No.	F_2 progenies	JJ803	S29742	RM1337	MRG4766	RM527	RM7311	Pb3810	RM224	Pi54
1	LR 5	A	A	A	A	A	A	A	A	A
2	SMS	B	B	B	B	B	B	B	B	B
3	F_2-1	B	A	H	A	A	NA	H	NA	H
4	F_2-2	A	H	H	H	A	H	B	B	B
5	F_2-3	H	H	H	H	A	H	A	A	H
6	F_2-4	H	A	H	A	NA	A	H	A	H
7	F_2-5	B	H	H	H	A	H	B	B	B
8	F_2-8	A	A	H	H	A	A	H	NA	NA
9	F_2-9	A	A	A	A	A	A	A	A	A
10	F_2-10	H	A	A	H	A	A	H	H	NA
11	F_2-13	H	A	H	A	A	A	A	A	A
12	F_2-14	A	H	H	B	B	H	B	B	B
13	F_2-15	H	A	A	B	A	A	B	B	B
14	F_2-16	B	A	H	H	A	NA	H	H	H
15	F_2-17	H	A	A	H	A	A	H	H	H
16	F_2-18	H	B	H	A	B	B	A	A	A
17	F_2-19	B	A	H	H	A	A	H	B	H
18	F_2-22	A	A	H	H	A	A	H	B	H
19	F_2-25	A	H	A	A	A	H	A	A	A
20	F_2-26	H	H	H	H	A	H	A	H	H
21	F_2-28	H	H	H	H	A	H	B	B	NA
22	F_2-29	H	H	H	H	A	H	NA	H	H
23	F_2-30	B	B	A	A	B	B	A	A	A
24	F_2-32	NA	H	H	B	A	H	B	B	B
25	F_2-34	A	A	H	H	A	A	H	A	A
26	F_2-35	B	B	A	NA	B	B	H	A	NA
27	F_2-37	H	A	H	H	A	A	B	H	H
28	F_2-39	A	H	H	A	B	H	A	A	A
29	F_2-40	H	A	A	A	A	A	A	A	A
30	F_2-42	A	A	H	H	A	A	A	H	NA
31	F_2-43	H	A	H	H	A	A	H	H	H
32	F_2-45	H	H	H	H	A	H	H	H	H
33	F_2-46	NA	B	H	H	B	B	H	H	NA
34	F_2-48	B	A	A	A	A	A	H	H	H
35	F_2-51	B	H	A	A	A	H	A	A	A
36	F_2-52	A	B	H	H	B	B	H	H	H
37	F_2-53	B	B	H	H	B	B	H	H	H
38	F_2-54	A	A	H	H	A	B	H	H	NA
39	F_2-57	NA	NA	NA	NA	NA	NA	NA	NA	NA
40	F_2-66	H	NA	H	A	A	H	A	A	A

41	F_2-70	B	NA	NA	H	A	A	H	H	NA
42	F_2-71	A	NA	NA	H	B	B	A	H	H
43	F_2-79	B	B	H	H	B	H	A	H	H
44	F_2-80	A	A	A	A	A	A	A	A	A

A-LR 5 allele, B- SMS allele, H-Heterozygous, NA-Didnot amplify

Table S5 Leaf and neck blast scores for the panel of rice genotypes

Label	Leaf Blast score	Neck Blast percentage	Genotype
1	5	100	LR26
2	0	0	JR19
3	4	67	BAM4534
4	0	79	IR2NIR20
5	0	0	DH1210
6	0	5	9W291
7	0	53	IRBL-b-R
8	3	22	4SHDH14
9	0	18	JR54
10	0	6	4SHDH14
11	0	0	BAM8296
12	4	0	BAM452
13	5	0	BAM4478
14	0	0	BAM5779
15	0	0	UR4
16	3	0	JR53
17	4	0	BAM4496
18	0	0	LR11
19	5	0	BAM3690
20	0	0	JR69
21	5	51	BAM5850
22	0	0	IRBLKK3
23	0	31	JR31
24	5	90	BAM7449
25	0	0	BAM2815
26	0	0	JR68
27	4	97	BLZ5CA
28	0	0	LR5
29	0	0	UR7
30	0	0	UR2
31	5	31	BAM4797
32	4	100	BAM8381
33	3	13	SMS
34	4	0	BAM7385
35	0	0	UR3
36	0	17	IRBL-5-6
37	4	0	BAM1529
38	3	42	BAM7859
39	0	0	UR84
40	0	0	UR01
41	3	38	BAM7429
42	0	0	BAM1659
43	0	0	JR60
